# The heterologous expression of a soybean (*Glycine max*) xyloglucan endotransglycosylase/hydrolase (XTH) in cotton (*Gossypium hirsutum*) suppresses parasitism by the root knot nematode *Meloidogyne incognita*

**DOI:** 10.1371/journal.pone.0235344

**Published:** 2020-07-06

**Authors:** Prakash M. Niraula, Katherine S. Lawrence, Vincent P. Klink

**Affiliations:** 1 Department of Biological Sciences, Mississippi State University, Mississippi State, Mississippi, United States of America; 2 Department of Entomology and Plant Pathology, Auburn University, Auburn, Alabama, United States of America; 3 Department of Biochemistry, Molecular Biology, Entomology and Plant Pathology, Mississippi State University, Mississippi State, Mississippi, United States of America; 4 Center for Computational Sciences High Performance Computing Collaboratory, Mississippi State University, Mississippi State, Mississippi, United States of America; USDA-ARS Southern Regional Research Center, UNITED STATES

## Abstract

A *Glycine max* (soybean) hemicellulose modifying gene, xyloglucan endotransglycoslase/hydrolase (XTH43), has been identified as being expressed within a nurse cell known as a syncytium developing within the soybean root undergoing the process of defense to infection by the parasitic nematode, *Heterodera glycines*. The highly effective nature of XTH43 overexpression in suppressing *H*. *glycines* parasitism in soybean has led to experiments examining whether the heterologous expression of XTH43 in *Gossypium hirsutum* (upland cotton) could impair the parasitism of *Meloidogyne incognita*, that form a different type of nurse cell called a giant cell that is enclosed within a swollen root structure called a gall. The heterologous transgenic expression of XTH43 in cotton resulted in an 18% decrease in the number of galls, 70% decrease in egg masses, 64% decrease in egg production and a 97% decrease in second stage juvenile (J2) production as compared to transgenic controls. The heterologous XTH43 expression does not significantly affect root mass. The results demonstrate XTH43 expression functions effectively in impairing the development of *M*. *incognita* at numerous life cycle stages occurring within the cotton root. The experiments reveal that there are highly conserved aspects of the defense response of *G*. *max* that can function effectively in *G*. *hirsutum* to impair *M*. *incognita* having a different method of parasitism.

## Introduction

The most abundant animals on earth are nematodes and among them are those that are parasitic to plants [1]. Plant parasitic nematodes present a significant problem for world agriculture, having been estimated to decrease world crop production by 7–10% or 0.1–0.2 trillion dollars, annually [2–4]. Therefore, identifying and developing strategies that would aid plants in defending themselves from parasitic nematodes through genetic resistance or other strategies are important.

The development of genetic resistance in plants to parasitic nematodes has been aided by a greater understanding of localized responses occurring at the site of nematode infection within the plant root. For example, RNA seq and microarray analyses of the infection site known as the syncytium, created through the parasitic activities of the soybean cyst nematode (SCN) (*Heterodera glycines*) in soybean (*Glycine max*), have led to the identification of many genes expressed specifically within those parasitized cells [5,6]. Among the genes whose expression was highly induced were homologs of the *Arabidopsis thaliana* BOTRYTIS INDUCED KINASE1 (BIK1) and xyloglucan endotransglycosylase/hydrolase (XTH) [6]. In *A*. *thaliana*, cytoplasmic BIK1 becomes phosphorylated through the activities of different microbe associated molecular patterns (MAMPs) on various membrane receptors. These receptors included FLAGELLIN SENSING2 (FLS2) and EF-Tu RECEPTOR (EFR) as well as DAMP PEPTIDE 1 RECEPTOR (AtPEPR1), each shared between BIK1 and BRI1-associated kinase 1 (BAK1) [7–10]. Furthermore, BIK1 can become autophosphorylated, leading to the activation of defense signaling processes [8,11,12]. Overexpression of *G*. *max* BIK1 was shown to suppress *H*. *glycines* parasitism in the susceptible genotype *G*. *max*_[Williams 82/PI 518671]_ [13]. In contrast, RNAi of BIK1 in the *H*. *glycines*-resistant genotype *G*. *max*_[Peking/PI 548402]_ impaired the defense response [13]. Overexpression of *G*. *max* BIK1 led to an increase in the expression of mitogen activated protein kinase 3 (MAPK3), which was shown to function in *G*. *max* resistance to *H*. *glycines* [14]. Furthermore, expression of MAPK3 in *G*. *max* led to the induction of XTH43 transcription [14]. These results provided a way to explain how the induction of XTH43 expression occurs. Unfortunately, it is unclear what role XTHs may have in defense responses for other plant species parasitized by different nematode species, such as the gall producing nematode *M*. *incognita* infecting *Gossypium hirsutum* (upland cotton).

XTH (EC 2.4.1.207) was found in all land plants and metabolizes the cell wall hemicellulose component xyloglucan [15–26]. Xyloglucan is a complex, branched polysaccharide with a (β1–4)-linked D-glucan backbone, tethering adjacent cellulose microfibrils through hydrogen bonding [27,28]. XTH functions in processes leading to cell enlargement during growth and also xyloglucan metabolism, including its biosynthesis, remodeling and organization [29,30]. XTH has been demonstrated to shorten xyloglucan chain length under certain conditions [31]. XTH exhibits the hallmarks of a secreted protein, having a signal peptide and traveling through the vesicle transport system to its targeted site of action [32]. This characteristic implies a functional vesicle transport system, including proteins functioning in vesicle and target membrane fusion, which is important to the delivery of XTH to its site of activity. This property of XTH likely explains why the major *H*. *glycines* resistance gene in *G*. *max* includes alpha soluble N-ethymaleimide-sensitive factor attachment protein (α-SNAP) which functions along with the soluble N-ethymaleimide-sensitive factor attachment protein receptor (SNARE) and *N*-ethylmaleimide-sensitive factor (NSF) in the fusion and recycling of SNARE proteins [33–35]. It also explains the observations of Bekal et al. (2015) who identified a secreted *H*. *glycines* protein that binds *G*. *max* α-SNAP [36], which would presumably impair the activities of SNARE by inhibiting the fusion of vesicle and target (cell) membranes. The ability of XTH to shorten xyloglucan chains may limit the expansion of cells that become incorporated into the syncytium, leading to an effective defense response [13]. Therefore, XTH may also function in impairing the parasitism of gall-producing nematode *M*. *incognita*.

The heterologous expression of genes identified in one plant (*G*. *max*) in another recipient plant (*G*. *hirsutum*) has been demonstrated to be an effective way to understand the genetics of defense in various plant-parasitic nematode pathosystems by generating resistance outcomes under conditions where they may not normally exist. For example, a number of *G*. *max* genes and various bacterial effectors have been tested in the *G*. *hirsutum*-*M*. *incognita* pathosystem showing that they function to impair parasitism [37–40]. Pant et al. (2016) showed that the *G*. *max* homolog of the *A*. *thaliana* transcription factor NONEXPRESSOR OF PR1 (NPR1) functions effectively to suppress *M*. *incognita* parasitism in *G*. *hirsutum* [37,41]. Furthermore, the bacterial effector harpin, first identified from the fire blight pathogen *Erwinia amylovora*, was capable of activating the expression of the coiled-coil nucleotide binding leucine rich repeat (CC-NB-LRR) NON-RACE SPECIFIC DISEASE RESISTANCE 1 (NDR1) in *G*. *max* [39,42–44]. The *A*. *thaliana* NDR1 functions through the MAPK signaling cascade to activate defense processes [42–49]. Harpin was shown to induce the expression of homologs of NDR1, specific MAPKs, NPR1 and XTH43, among other genes in the *G*. *max*-*H*. *glycines* pathosystem [14,39,40]. The induction of NDR1 can function in *G*. *max* on different pathogens, including the charcoal rot ascomycete (*Macrophomina phaseolina*), indicating a broad effectiveness for different types of pathogens [50]. Heterologous expression approaches were also used to examine these *G*. *max* genes in *G*. *hirsutum* to determine early signaling events that are transduced either through pathogen effectors (harpin), membrane receptors (NDR1), or transcription factors (NPR1) [14,38–40]. However, these experiments have not taken into consideration the subsequent products of these signaling events including genes like XTH that were induced by harpin, NDR1, BIK1, NPR1 and MAPKs [13,14,39,40].

Therefore, the objective of the present study was to examine the downstream component of the XTH defense process because the defense gene XTH43 performs a very specific role in cell wall metabolism [20,31]. The heterologous expression of the *G*. *max* XTH43 in *G*. *hirsutum* was evaluated to determine whether it can interfere with the life cycle of *M*. *incognita*. XTH43-expressing *G*. *hirsutum* roots infected with *M*. *incognita* were evaluated for their effect on galls, egg masses, eggs and second stage juveniles (J2s). The results showed a negative impact on *M*. *incognita* parasitism, but that effect was more apparent during the life cycle when egg masses, eggs and J2s are produced.

## Materials

### Gene selection and production of genetically mosaic transgenic *G*. *hirsutum* plants

The 864 bp *G*. *max* xyloglucan endotransglycosylase/hydrolase 43 (XTH43) (Glyma.17G065100) sequence has been expressed in *G*. *hirsutum* to evaluate its effect on *M*. *incognita* parasitism using published methods [37]. The Gateway^®^-compatible pRAP15 overexpression vector has the *ccd*B gene that is lethal to *E*. *coli*, functioning as an effective chemical selectable marker during vector engineering (Invitrogen) [51,52]. The XTH43 gene has been directionally engineered into the pRAP15 vector [13]. The pRAP15-XTH43 plasmids have been genetically transformed into *Agrobacterium rhizogenes* strain 15834 (15834) and selected on LB-agar supplemented with 5 μg/ml tetracycline [37]. Seeds of *M*. *incognita*-susceptible *G*. *hirsutum* (Phytogen 565 WRF) were planted in pre-wetted sterilized sand for germination. The seedlings were grown for 14 days at ambient greenhouse temperatures (~26-29°C). The seedlings were then removed from the sand and washed in sterile, deionized water. The root was excised with a sterile razor blade. *G*. *hirsutum* have been genetically transformed as described by McNeece et al. (2017) [40]. Briefly, an overnight culture of 15834 was grown in YEB Agrobacterium Growth Medium (Bioworld) according to the manufacturer’s instructions and supplemented with 5 μg/ml tetracycline. The 15834 culture was then pelleted during a 20 min spin at 4,000 RPM in a Sorvall RC6+ centrifuge and the pellet was re-suspended in 25 mL of Murashige and Skoog (MS) media including vitamins (Duchefa), pH 5.7 [40]. Subsequently, 25 root-less *G*. *hirsutum* plants per group were placed in a 140 ml beaker containing 25 ml of 15834 harboring the pRAP15-XTH expression plasmid or pRAP15-*ccd*B control. The plants were placed under a vacuum for 20 minutes. After 20 minutes, the vacuum was slowly released over a period of 5 minutes. The root-less *G*. *hirsutum* plants were then placed in 50 cell flats (T.O Plastics) with one plant per cell in coarse A-3 vermiculite (Palmetto Vermiculite). The 50-cell flats were placed in 24 L, 61.9 X 34.8 X 15.6 cm plastic containers (Sterlite) with the lid secured for 2 weeks while the plants recover under fluorescent lights. The recovered plants are subsequently placed in a greenhouse under ambient temperatures for two weeks prior to selection of transgenic plants.

The visual selection of transgenic roots has been accomplished with the enhanced green fluorescent protein (eGFP) reporter system [53]. The pRAP15 vector, containing the eGFP reporter is used to generate expression of a targeted gene [40,52,54,55]. In control and experimental plants, the eGFP and control or XTH43 genes will each have their own promoter and terminator sequences [13,37,40,52,55]. Due to the manner that 15834 transfers the DNA cassettes located between the left and right borders of the destination vector into the root cell chromosomal DNA, the subsequent growth and development of the stably transformed genetically engineered cell into a transgenic root results in the production of a plant that is a genetic mosaic called a composite plant [55,56]. These genetically mosaic plants have the entire shoot being non-transgenic while the entire root system is transgenic [55–57]. Consequently, each individual transgenic root system functions as an independent transformant line [13,40,52,56,58].

### cDNA synthesis

*G*. *hirsutum* root mRNA has been isolated using the UltraClean^®^ Plant RNA Isolation Kit (Mo Bio Laboratories^®^, Inc.) according to the manufacturer’s instructions. Genomic DNA has been removed from the mRNA with DNase I (Invitrogen^®^) according to the manufacturer’s instructions. The cDNA has been synthesized from mRNA using the SuperScript^®^ First Strand Synthesis System for RT-PCR (Invitrogen^®^) according to the manufacturer’s instructions. Oligo d(T) has been selected as the primer (Invitrogen^®^) for cDNA production with the experimental procedure performed according to the manufacturer’s instructions.

### PCR

The presence of the XTH43 RNA has been demonstrated in PCR experiments that used cDNA produced from transgenic *G*. *hirsutum* root RNA. The quantitative real time PCR (qRT-PCR) control used in the *G*. *hirsutum* experiments has been designed from *Gossypium raimondii* S21 (Gorai.009G233700.1) (S1 Table) (Pant et al. 2015). Confirmation of eGFP expression has been performed by qRT-PCR according to McNeece et al. (2017) using Taqman^®^ 6-carboxyfluorescein (6-FAM) probes and Black Hole Quencher (BHQ1) (MWG Operon; Birmingham, AL). Fold change has been calculated using 2^-ΔΔ*C*^_T_ [59] according to published methods [40]. PCR using cDNA produced from mRNA isolated from the pRAP15 control and the XTH43-expressing roots has been used to demonstrate that XTH43 has been expressed in the transgenic roots. The qRT-PCR data has been analyzed statistically using the Student’s *t*-test.

### Infection by *M*. *incognita* and analysis of results

The *M*. *incognita* (race 3) have been confirmed by the North Carolina differential host test and increased on *Lycopersicum esculentum* (tomato) under ambient greenhouse conditions [60–64]. [Eggs were extracted from roots by placing the root system in a 0.625% NaOCl solution and agitating the roots for 4 min using a rotary shaker at 120 rpm Eggs were rinsed with tap water, collected on a 25-μm-pore sieve, then processed by sucrose centrifugation-flotation at 240 g for 1 minute [60]. *M*. *incognita* eggs were placed in a modified Baermann funnel [65] on a slide warmer (Model 77) (Marshall Scientific, Brentwood, NH) and incubated at 31°C for 5 to 7 days to obtain second stage juveniles (J2) [66]. The J2 were collected on a 25-μm-pore sieve, transferred to 1.5 ml microcentrifuge tubes, centrifuged at 5,000 g for 1 minute, rinsed with sterile distilled water, and centrifuged at 5,000 g for 1 minute. The J2 suspension was adjusted to 30 to 40 J2 per 10 μl of water [66]. *M*. *incognita* extraction has been performed by gravity screening and centrifugal flotation (sucrose specific gravity = 1.13) [60]. *M*. *incognita* eggs and second stage juveniles (J2s) were extracted from *L*. *esculentum* roots by a 4 minute root immersion in 0.525% NaOCl [61]. The hatched *M*. *incognita* J2s have been maintained at 4 ± 1 °C in water at until inoculation [63].

Transgenic *G*. *hirsutum* plants have been grown in 15 cm diameter clay pots. The pots were filled with 500 cm^3^ of the sterilized soil-sand mixture. In these pots, a suspension of 2,500 *M*. *incognita* in 3 ml suspension were pipetted into each of two holes (2.5 cm diameter x 2.5 cm deep). The plants grew in the greenhouse maintained at a temperature range of 25° to 35°C given at least 12 hours/day of ambient light. The *M*. *incognita* life-stage development has been described using a modified Christie’s method [63,67,68]. The nematodes were extracted by combined gravity screening and sucrose centrifugation at 50 days post infection (dpi). The nematodes were then enumerated on grated Petri dishes with an Olympus BH2 B071 microscope (Japan Model C35AD-4) at 40 X magnification [39]. Root fresh weights have also been determined to allow the calculation of galls, egg masses, eggs and J2s per gram of root tissue [38]. The enumeration and statistical analyses of galls, egg masses, eggs and J2s have been done, standardizing them in two different ways in order to differentiate between the effects that XTH43 expression has on *M*. *incognita* development in relation to the root. These two different analyses include enumerating the number of galls, egg masses, eggs and J2s in the whole root (wr) and per gram (pg) of root tissue. In each analysis the results are considered statistically significant if p < 0.05, determined using Mann-Whitney-Wilcoxon Rank Sum Test.

## Results

### Generation of transgenic plants heterologously expressing XTH43

**Fig 1:**
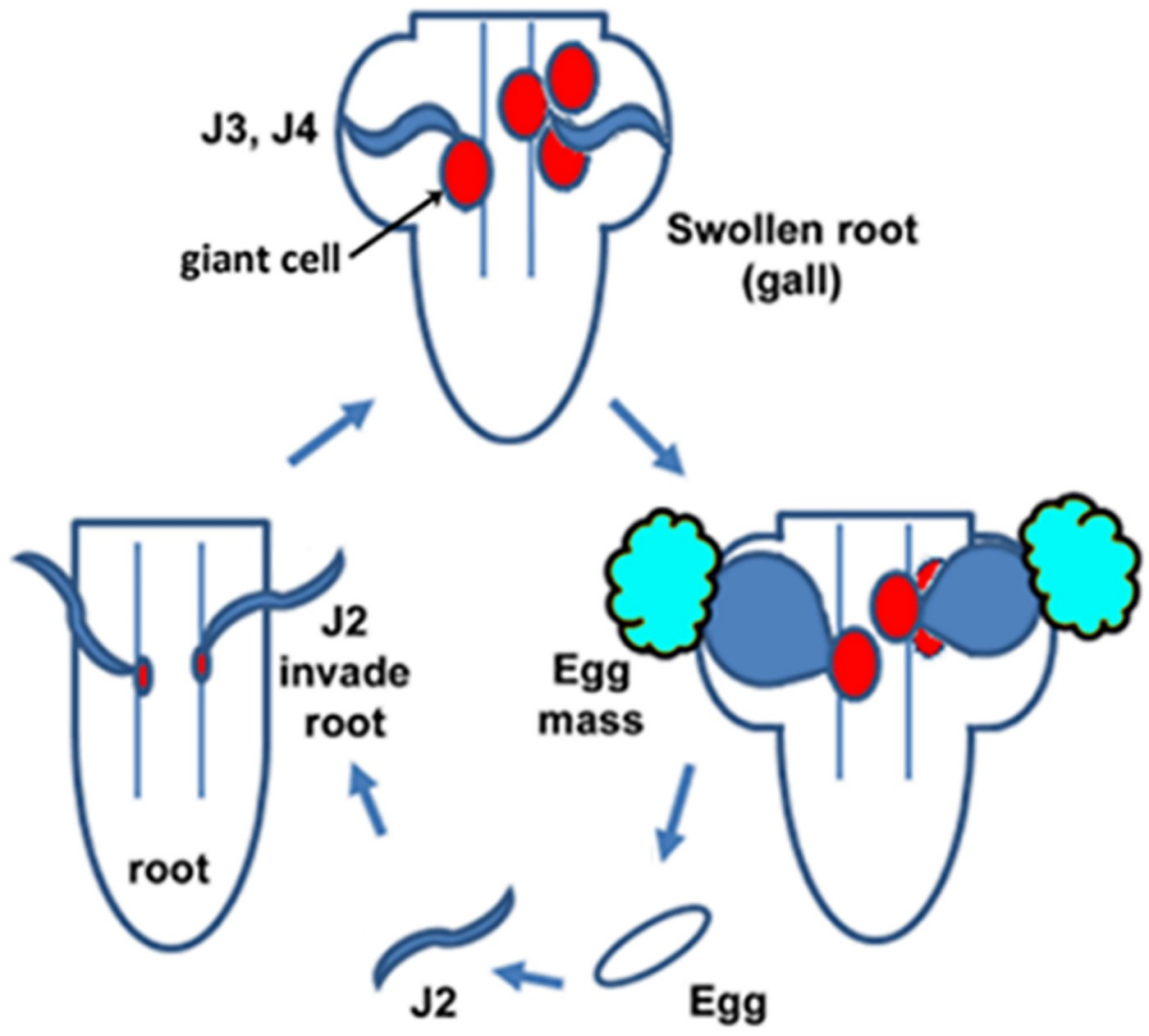
*M*. *incognita* life cycle showing egg, J2, J3, J4, females with egg masses, giant cell and swollen root (gall).

**Fig 2:**
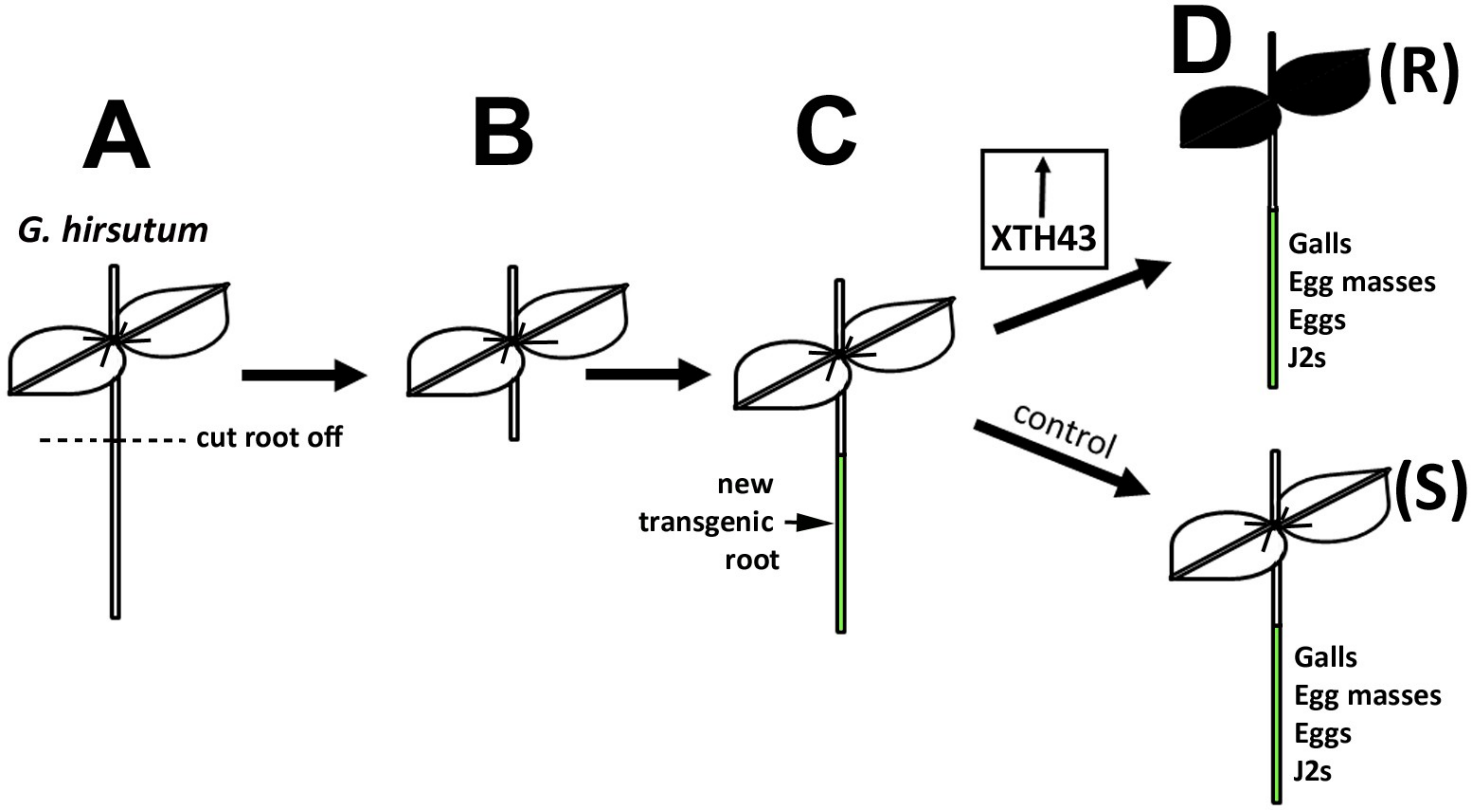
Transgenic strategy. **A**. *G*. *hirsutum* will have its root removed (dashed line). **B**. *G*. *hirsutum* with its root removed. **C**. A new transgenic root grows. **D**. One cache of roots is engineered to express the *G*. *max* XTH43. Another cache of roots is genetically engineered with pRAP15-*ccd*B, lacking XTH43. The roots, after infection for 50 days are analyzed for galls, egg masses, eggs and J2s. (R), engineered resistant reaction (XTH43-expressing); (S) susceptible reaction (control).

**Fig 3:**
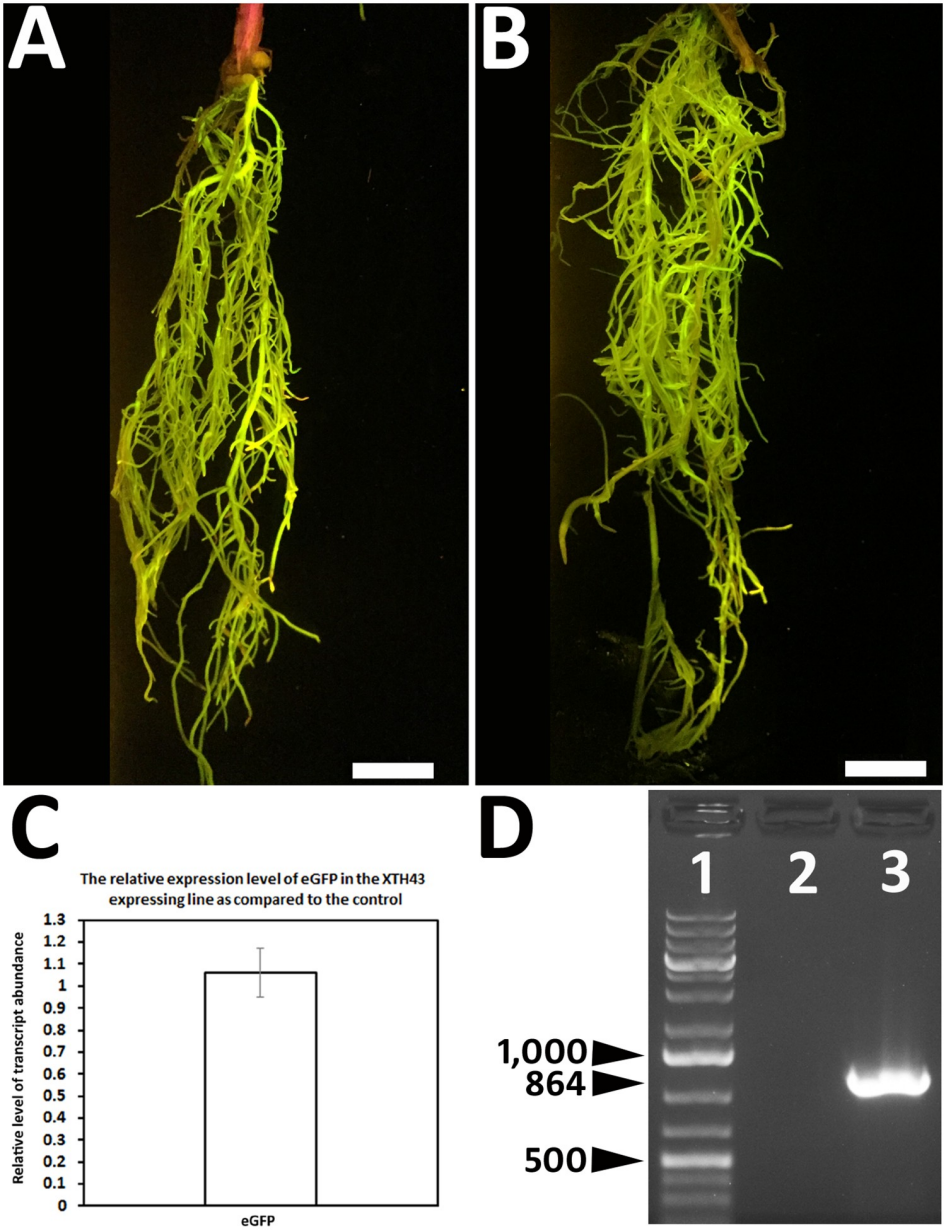
Generation of transgenic roots. **A**. pRAP15 control root revealed by the eGFP reporter. Bar = 1 cm. **B**. XTH43-expressing engineered root revealed by the eGFP reporter. Bar = 1 cm. **C**. qRT-PCR demonstrates similar expression of the eGFP reporter construct as compared to the S21 control is occurring at a similar level between the pRAP15 control and XTH43-expressing transgenic lines. **D**. PCR has been used to demonstrate the presence of the XTH43 transcript only in the transgenic XTH43 expressing line in comparison to the control; lane 1, DNA ladder with base pairs indicated with black arrowheads; lane 2, control; lane 3, XTH43-expressing line.

Transgenic approaches in *G*. *hirsutum* allow for an analysis of the effect that the expression of the XTH43 transgene has on different aspects of *M*. *incognita* life cycle. Among these features are the development of galls, production of egg masses, eggs and J2s (Fig 1). *G*. *hirsutum* plants have had their roots excised with transgenic roots subsequently regrown for the purposes of the experimental approach and goals (Fig 2). Genetically mosaic *G*. *hirsutum* plants having transgenic roots and a non-transgenic shoots showed eGFP fluorescence in the roots for pRAP15-*ccd*B (control) and XTH43 (experimental) lines, demonstrating a functional reporter while PCR confirmed XTH43 expression (Fig 3). The number of control plants and XTH43-expressing transgenic plants spanning three biological replicates were examined for the *M*. *incognita* infection response presented in the subsequent studies (Table 1).

**Table 1:** The number of transgenic plants analyzed in the study.

	Control	XTH43 expressing
**Replicate 1**	**22**	**23**
**Replicate 2**	**24**	**22**
**Replicate 3**	**26**	**25**
**Total**	**72**	**70**

### Effect of XTH43 expression on *M*. *incognita* development

**Fig 4:**
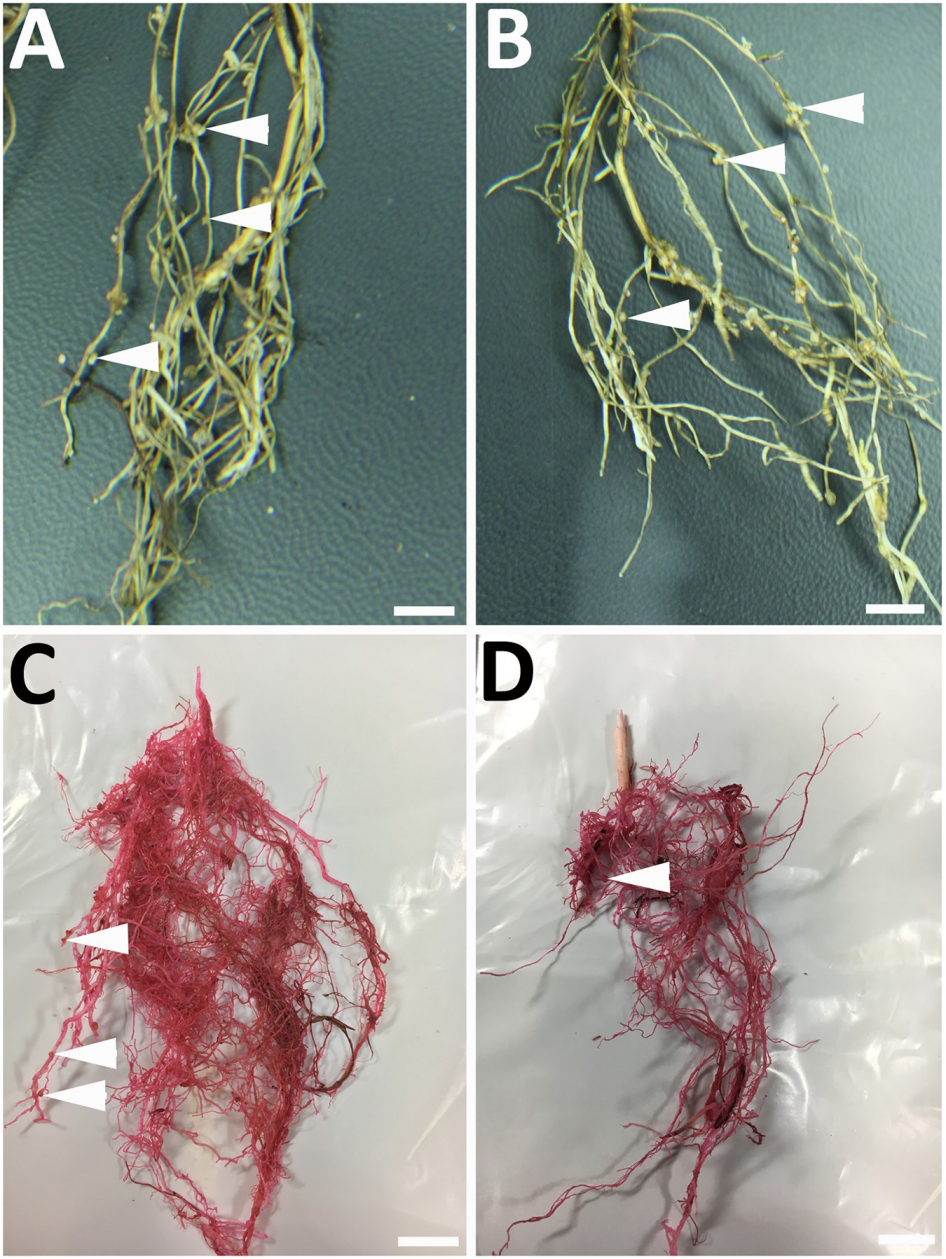
Morphological features of *M*. *incognita* infection of *G*. *hirsutum*. **A**. Root galls in pRAP15-control. **B**. root galls in XTH43-expressing *G*. *hirsutum*. **C**. Egg masses in pRAP15-control. **D**. Egg masses in XTH43-expressing *G*. *hirsutum*. White arrowheads point to galls (**A**, **B**) or egg masses (**C**, **D**). Bars = 1 cm.

**Fig 5:**
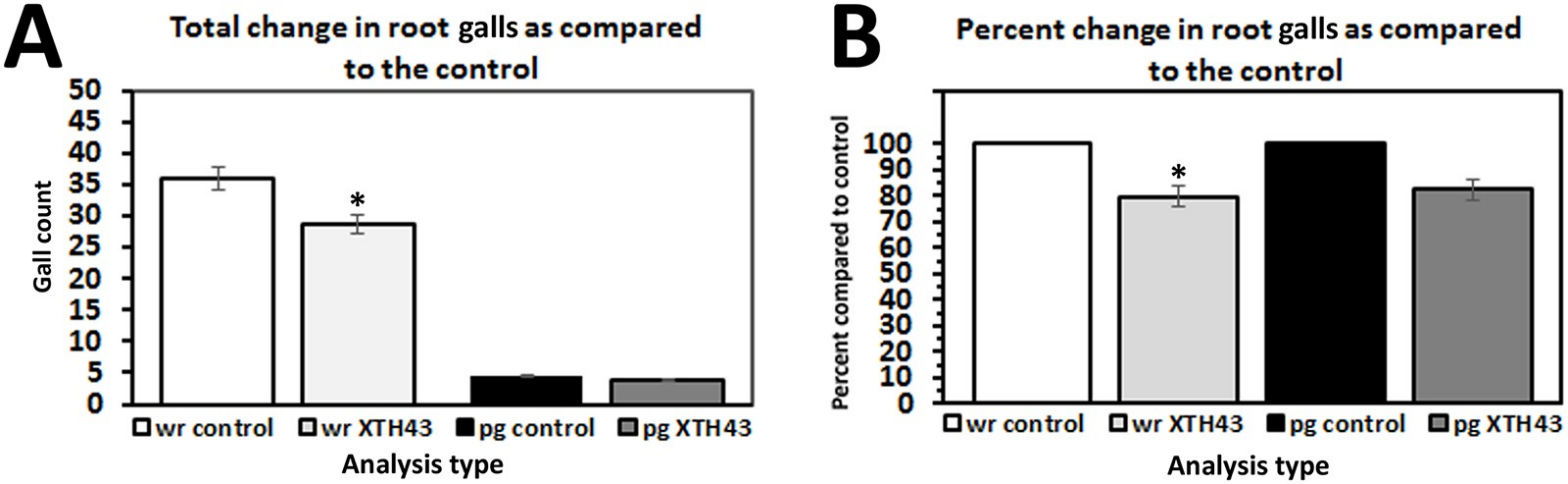
*M*. *incognita*-induced root gall analyses in whole roots (wr) and per gram (pg) of root tissue show *G*. *hirsutum* roots genetically engineered to express XTH43 affects their parasitism. **A**. Total change in root galls as compared to the control (wr, p = 0.046*; pg, p = 0.234). **B**. Transformed data from A showing percent change in root galls as compared to the control (wr, p = 0.046*; pg, p = 0.234). (*) denotes statistically significant p < 0.05, determined using Mann-Whitney-Wilcoxon Rank Sum Test.

The analysis of the effect of XTH43 expression in *G*. *hirsutum* began by examining the number of galls produced by *M*. *incognita*, followed by analyses of the numbers of egg masses (Fig 4). The total number of galls for the whole root evaluation showed a mean of 28.6 galls for the XTH43-expressing plants, which was a significant decrease (p = 0.046) of 20.4% compared to a mean of 35.9 galls for the control plants (Fig 5). However, the mean number of galls per gram of root tissue for the treatments were not significant (p = 0.234). XTH43-expressing roots showed a mean of 3.7 galls per gram of root compared to a mean of 4.5 galls per gram of root for the control, which was a 17.7% reduction (Fig 5).

### Effect of XTH43 expression on egg mass development

**Fig 6:**
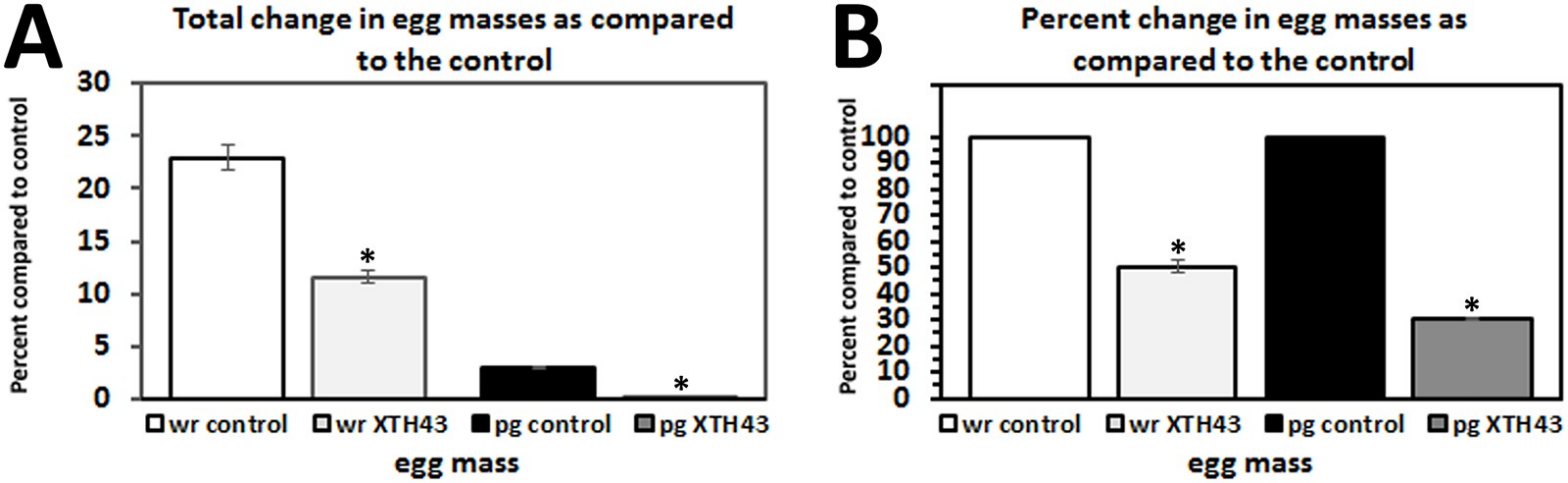
*M*. *incognita* egg mass analyses in whole roots (wr) and per gram (pg) of root tissue show *G*. *hirsutum* roots genetically engineered to express XTH43 affects their parasitism. **A**. Total change in egg masses as compared to the control (wr, p = 0.002*; pg, p = 0.012*). **B**. Transformed data from A showing percent change in egg masses as compared to the control (wr, p = 0.002*; pg, p = 0.012*). (*) denotes statistically significant p < 0.05, determined using Mann-Whitney-Wilcoxon Rank Sum Test.

The total number of egg masses in XTH43-expressing roots was significantly decreased (p = 0.002). Roots of XTH43-expressing plants show a 49.6% reduction egg masses with a mean of 11.6 egg masses compare to a mean of 22.9 egg masses for the control plants (Fig 6). The number of egg masses per gram of root also showed a significant reduction (p = 0.012) with a mean of 0.09 for XTH43-expressing plants and a mean of 3.0 for control plants, which represents a 70% reduction in nematode reproduction (Fig 6).

### Effect of XTH43 expression on egg development

**Fig 7:**
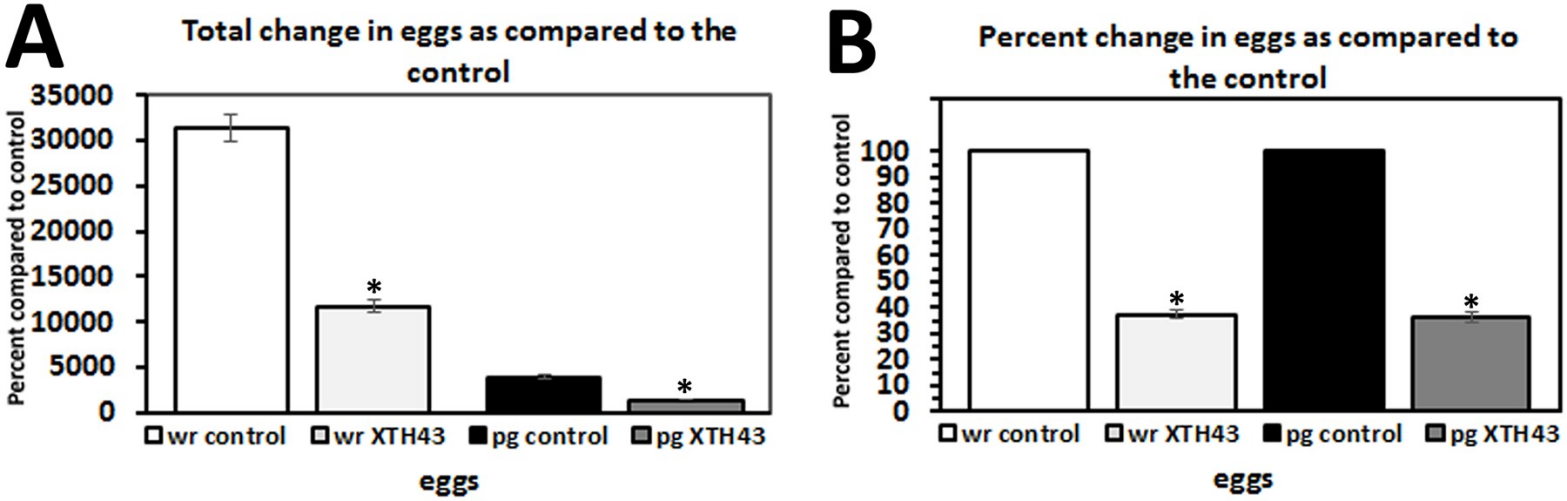
*M*. *incognita* egg analyses of in whole roots (wr) and per gram (pg) of root tissue show *G*. *hirsutum* roots genetically engineered to express XTH43 affects their parasitism. **A**. Total change in eggs as compared to the control (wr, p < 0.001*; pg, p < 0.001*). **B**. Transformed data from A showing percent change in eggs as compared to the control (wr, p < 0.001*; pg, p < 0.001*). (*) denotes statistically significant p < 0.05, determined using Mann-Whitney-Wilcoxon Rank Sum Test.

A mean of 11,748.4 eggs were produced on roots of XTH43-expressing plants and 31,382.8 eggs for control plants with a mean of 1,414.9 eggs per gram of root for XTH43-expressing plants and a mean of 3,892.1 eggs per gram of root for the control plants (Fig 7). These data represent a significant decrease in the total number of eggs (p < 0.001) and number of eggs per gram of root (p < 0.001) with an overall decrease of approximately 62.6% and 63.6% for the two analyses, respectively.

### Effect of XTH43 expression in *G*. *hirsutum* on J2 development

**Fig 8:**
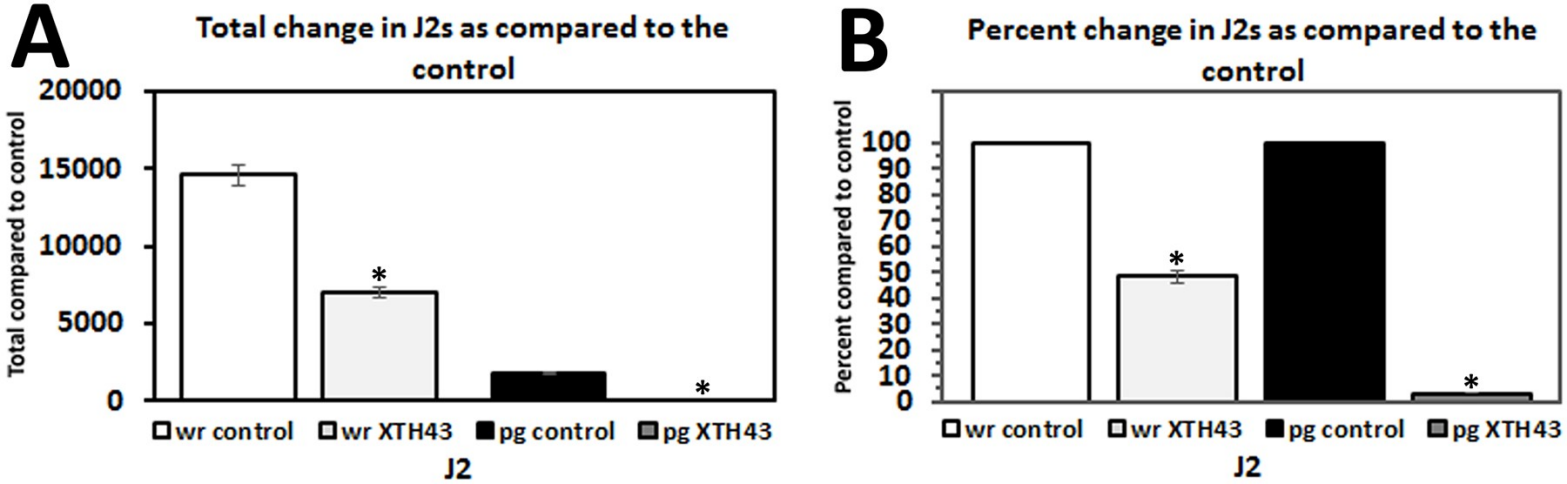
*M*. *incognita* J2 analyses in whole roots (wr) and per gram (pg) of root tissue show *G*. *hirsutum* roots genetically engineered to express XTH43 affects their parasitism. **A**. Total change in J2s as compared to the control (wr, p < 0.001*; pg, p = 0.007*). **B**. Transformed data from A showing percent change in juveniles as compared to the control (wr, p < 0.001*; pg, p = 0.007*). (*) denotes statistically significant p < 0.05, determined using Mann-Whitney-Wilcoxon Rank Sum Test.

XTH43-expressing plants showed a significant reduction in J2s for the whole root evaluation and for the evaluation based on root weight. For the whole root evaluation, a mean of 7,049.1 J2s were recorded for XTH43-expressing plants and a mean of 14,572.9 J2s for the control plants representing a statistically significant reduction of 62.6% (p < 0.001) (Fig 8). A mean of 55.9 J2s per gram of root was observed for the XTH43-expressing plants compared to a mean 1,758.6 for the control plants resulting in a statistically significant reduction of 96.8% (p = 0.007) (Fig 8).

### Effect of XTH43 expression in *G*. *hirsutum* has on root mass

**Fig 9:**
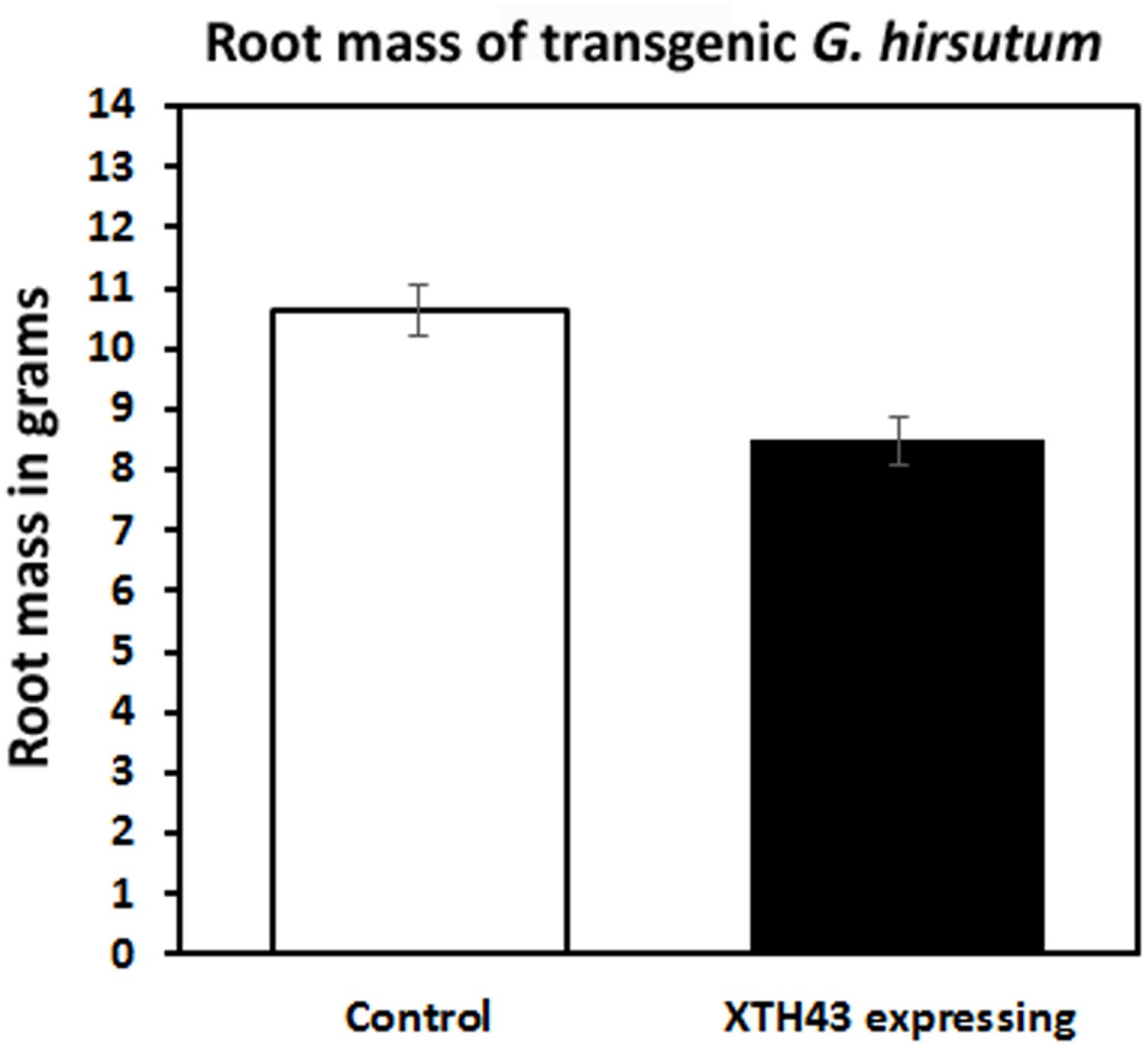
Root masses of transgenic plants used in the analysis. The control root mass has been determined to be 10.631 g as compared to 8.494 g in the XTH43 expressing roots, p = 0.897 determined using Mann-Whitney-Wilcoxon Rank Sum Test.

An analysis was done to determine if XTH43 expression in *G*. *hirsutum* had any impact on root mass. The XTH43 expressing plants showed a 20.1% decrease in root mass compared to the control (Fig 9). However, this decrease was not significant (p = 0.897).

## Discussion

The heterologous expression of the *G*. *max* XTH43 in *G*. *hirsutum* has been examined here to determine if it would suppress *M*. *incognita* development because it functions effectively in soybean to suppress *H*. *glycines* parasitism [13]. The heterologous expression of XTH43 in *G*. *hirsutum* does not appear to lead to any statistically significant adverse effect on root mass. There is approximately an 18–20% decrease in the number of galls as a consequence of XTH43 expression in *G*. *hirsutum* indicating XTH43 has a slight effect on earlier stages of parasitism. However, other aspects of the *M*. *incognita* life cycle have been shown here to be more sensitive to XTH43 expression in *G*. *hirsutum* with almost a 97% reduction in J2s, ranking it among the most effective heterologously expressed *G*. *max* genes on *M*. *incognita* parasitism.

### A relationship between the *G*. *max* XTH43 and the *G*. *hirsutum* genome

Blast searches of the *G*. *hirsutum* cultivar TM-1 using the *G*. *max* XTH43 protein sequence were performed after obtaining the results that show that the *G*. *max* XTH43 expression negatively effects *M*. *incognita* development. Those unpublished results that are not a part of these experiments, revealed XTH43 was 82% identical to LOC107907479 (NCBI identifier XP_016690364). There were 14 other *G*. *hirsutum* protein sequences having between 69–81% identity to XTH43 and even more having identities that were much lower. Our prior experiments have revealed that the *G*. *max* XTH43 occurs among a gene family having 53 total homologs [13]. XTH43 exists on chromosome 17 in a region of DNA having a localized amplification in gene copy number [13]. This region of DNA has 6 copies of XTH (XTH41, XTH42, XTH43, XTH44, XTH45, XTH46) [13]. In *G*. *max*, copy number variation appears to play a role in defense to *H*. *glycines* under certain circumstances. However, what is more clear is the importance of the level of expression of those genes to the defense process [13,52]. An examination of the *G*. *hirsutum* XTH gene family was beyond the scope of the analysis here. However, the findings provide important insight into the design of experiments that could lead to the demonstration of specialized functions for specific XTH genes that function in defense. These would be important experiments, moving forward.

### Model

**Fig 10:**
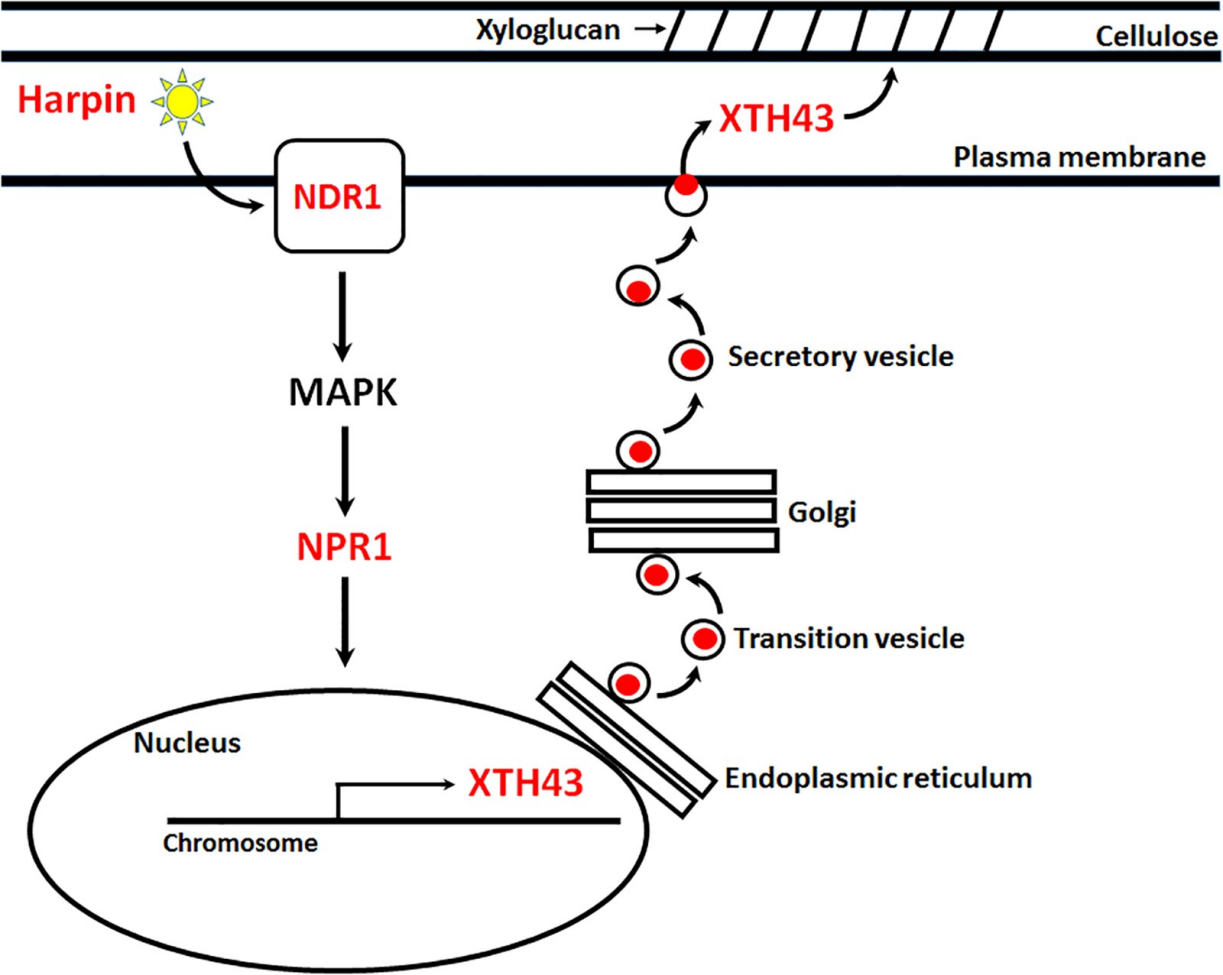
Model. The bacterial effector harpin has been shown to suppress *M*. *incognita* parasitism in *G*. *hirsutum* and function to induce the expression of NDR1 (Aljaafri et al. 2017). NDR1 expression leads to induced MAPK and NPR1 expression (McNeece et al. 2017). NDR1, MAPK and NPR1 expression all induce XTH43 expression (McNeece et al. 2019). The heterologous expression of NDR1 and NPR1 in *G*. *hirsutum* functions to decrease *M*. *incognita* parasitism (Pant et al. 2016; McNeece et al. 2017). Genes in red, and harpin, have been demonstrated, when expressed heterologously in *G*. *hirsutum* or topically applied, to impair *M*. *incognita* parasitism (Matsye et al. 2012; Pant et al. 2015; Aljaafri et al. 2017; Sharma et al. 2016; McNeece et al. 2017; Lawaju et al. 2018).

Over the past few years, a series of genes that function in *G*. *max* in its defense to *H*. *glycines* have been shown through their heterologous expression to impair *M*. *incognita* parasitism in *G*. *hirsutum* [13,14,35,37,38,40]. Importantly, the analyses have included an examination of *G*. *max* homologs of NDR1, as well as the harpin effector that induces NDR1 expression (Fig 10) [39]. Prior studies have shown that expression of the *G*. *max* NDR1 in *G*. *hirsutum* reduces *M*. *incognita* gall production by 65.2% [40] as compared to the 17.7% reduction found here by XTH43 expression. These results are consistent with the observation that the induction of *G*. *max* NDR1 expression affects other cellular processes [40]. For example, the overexpression of the *G*. *max* NDR1 leads to an increase in expression of a number of defense genes other than those already described (NPR1, BIK1, XTH43). Among those genes are components of the 20S particle, including the *rhg1* gene α-SNAP, NSF, syntaxin31, syntaxin121, synaptobrevin, synaptotagmin, mammalian uncoordinated 18 (Munc18), SNAP25 and NSF [40]. Among NDR1-induced genes associating with SA signaling include NPR1, ENHANCED DISEASE SUSCEPTIBILITY1 (EDS1), TGA2 and LESION SIMULATING DISEASE 1 (LSD1) [40]. Among the NDR1-induced genes are also those associating with glucoside production, including α-hydroxynitrile glucosidase and cytochrome P450 (CYP) 79D4 (CYP79D4) [40]. Furthermore, an ATP binding cassette (ABC) transporter has been identified as being induced by NDR1 [40]. Lastly, NDR1 induces the expression of a gene shown to function additively with *rhg1* (α-SNAP), the serine hydroxymethyltransferase (*Rhg4*) [40].

In contrast to these observations, it is possible the events that are important for defense and require XTH43 are impaired by the parasitic activities of *M*. *incognita*. For example, Yokoyama et al. (2001) have shown that XTH travels through the vesicle transport system to be delivered for its role in hemicellulose modification [32] and as stated, Bekal et al. (2015) have identified a secreted *H*. *glycines* protein that binds *G*. *max* α-SNAP, which could impair SNARE [36]. In comparisons of *G*. *max* NDR1, its expression leads to a 68.8% decrease in egg mass production [40] in comparison to a 97.1% decrease in *G*. *hirsutum* roots expressing XTH43. It appears that heterologous expression of XTH43 effectively impairs *M*. *incognita* parasitism in the later stages. In experiments examining egg production, the expression of *G*. *max* NDR1 leads to a 78.5% decrease in *M*. *incognita* egg production [40]; whereas, the decrease in *M*. *incognita* egg production was lower in roots expressing XTH43. Lastly, the expression of the *G*. *max* NDR1 leads to a 97.3% decrease in *M*. *incognita* J2 production [40]. This outcome is similar to a 96.8% decrease in *M*. *incognita* J2 production in roots expressing XTH43. A number of experiments have shown that it is possible to examine components of a defense response to parasitic nematodes that is induced by the bacterial effector harpin [39]. Harpin has been shown to induce the expression of genes that relate to important aspect of genetic resistance in *G*. *max*. Furthermore, the observation that harpin induces *rhg1* is consistent with the role that the vesicle transport process has to transport cell wall modifying proteins like XTH43. Once at the apoplast, XTH43 would be in position to alter the hemicellulose by modifying xyloglucan chain length. *Rhg4* functioning in 1-carbon metabolic processes could be employed to redirect carbon to the production of xyloglucan for cell wall building and modifying processes that impair the life cycle of parasitic nematodes [69]. However, regarding harpin, recent experiments have shown it is susceptible to deactivation by bacterial effectors so this issue must be taken into consideration in any work regarding harpin [70]. The results presented here demonstrate the function of XTH43 in impairing parasitic nematodes is likely to be conserved. Furthermore, its association with the vesicle transport machinery make it feasible to believe that a number of conserved cellular processes are employed to facilitate its transport and activity. The results also show that the genetic transfer of defense components from one plant to another can be activated and function to a very high level of fidelity, overcoming the influence of the pathogen. The results presented here are of significant interest to determine ways to develop resistance to pathogens where is normally does not exist.

## Supporting information

S1 Table PCR and qRT-PCR primers. (XLSX)

S1 Raw images (PDF)
