# Supplementary material for: The heterologous expression of a soybean (Glycine max) xyloglucan endotransglycosylase/hydrolase (XTH) in cotton (Gossypium hirsutum) suppresses parasitism by the root knot nematode Meloidogyne incognita
Source: PLoS One. 2020 Jul 6;15(7):e0235344. doi: 10.1371/journal.pone.0235344 (PMC7337317; doi:10.1371/journal.pone.0235344)

Supplement to the gel image in Figure 3. Red "X" represents extraneous lanes relating to other experiments that are not part of the present work. 1, DNA ladder; lane 1, DNA ladder with the red arrow pointing to 1,000 bp; lane 2, control; lane 3, XTH43-expressing line.

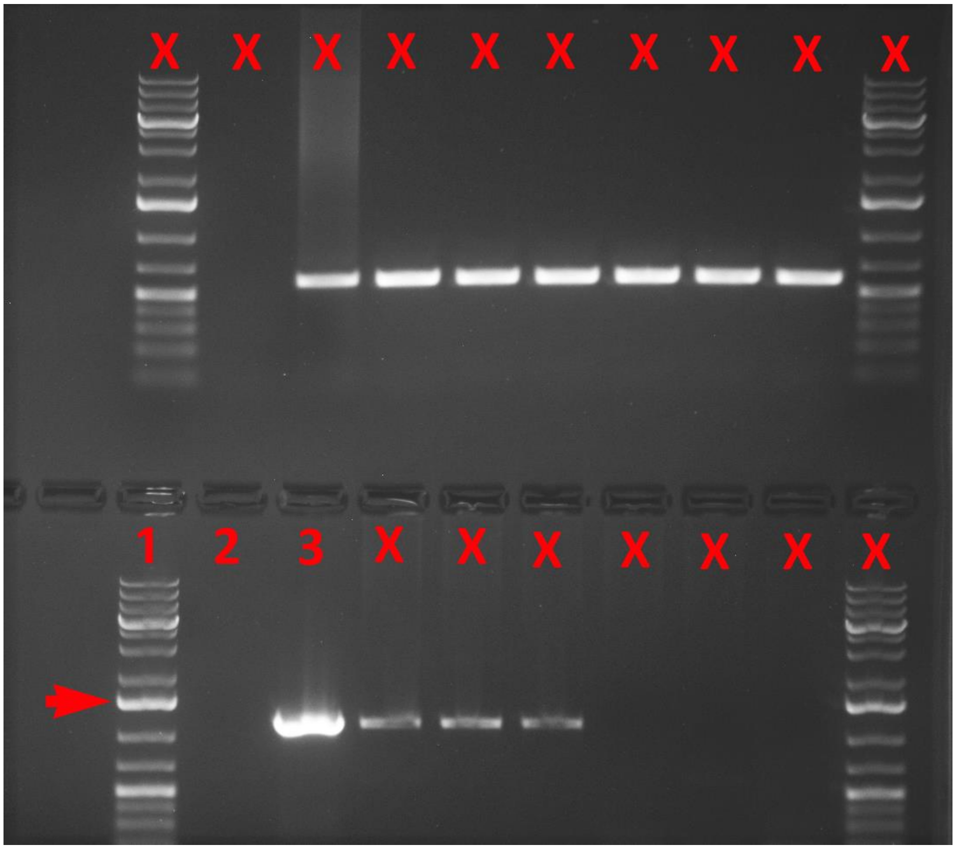

Supplement: S1 Raw images — (PDF) [file pone.0235344.s002.pdf]
